# The Prognostic and Predictive Roles of Ataxia–Telangiectasia Mutated (ATM) Expression in Patients with Metastatic Non-Small-Cell Lung Cancer Receiving Pembrolizumab Monotherapy Alone or in Combination with Chemotherapy

**DOI:** 10.3390/diagnostics15081048

**Published:** 2025-04-21

**Authors:** Jamshid Hamdard, Harun Muğlu, Ahmet Bilici, Elif Kuzucular, Özgür Açıkgöz, Ömer Fatih Ölmez, Oktay Olmuşçelik, Özcan Yıldız

**Affiliations:** 1 Medical Oncology Department, Faculty of Medicine, Medipol University, İstanbul 34214, Türkiye; hm1635@hotmail.com (H.M.); ahmetknower@yahoo.com (A.B.); ozgur_acikgoz@yahoo.com (Ö.A.); olmezof@gmail.com (Ö.F.Ö.); ozcanyildiz71@gmail.com (Ö.Y.); 2 Medical Pathology Department, Faculty of Medicine, Medipol University, İstanbul 34214, Türkiye; elifcalis261@gmail.com; 3 Internal Medicine Department, Faculty of Medicine, Medipol University, İstanbul 34214, Türkiye; oolmuscelik@medipol.edu.tr

**Keywords:** non-small-cell lung cancer, ATM expression, pembrolizumab, progression-free survival, overall survival

## Abstract

**Background/Objectives:** This study investigated the prognostic and predictive significance of Ataxia–Telangiectasia Mutated (ATM) expression in patients with metastatic non-small-cell lung cancer (NSCLC) who were treated with pembrolizumab. **Methods:** A retrospective analysis was conducted on 49 patients with metastatic NSCLC who received first-line pembrolizumab, either as a single agent or in combination with chemotherapy. ATM expression in archival pathology specimens was assessed using immunohistochemistry, where nuclear staining was considered to be positive. ATM expression was categorized into low- and high-expression groups based on staining intensity and the percentage of positive cells. Subsequently, the prognostic and predictive value of ATM expression was evaluated. **Results:** In terms of demographics, the mean age was 62.7 ± 9.5, most patients (91.8%) were male, and the majority (75.5%) had adenocarcinoma. The objective response rate (ORR) was 69.4%, and ATM expression was high in 75.5% of patients. Patients with low ATM expression had significantly longer progression-free survival (PFS) compared to those with high expression (51 vs. 5.7 months, *p* = 0.004). In multivariate analysis, ATM expression was the only independent prognostic factor for PFS, showing that patients with high ATM expression had a shorter overall survival (OS) compared to those with low expression (51 vs. 8.9 months, *p* = 0.013), which was statistically significant (HR 2.41, *p* = 0.034). Logistic regression analysis showed that ATM expression, as well as the presence of bone metastasis and the absence of liver metastasis, was significantly associated with a response to treatment (*p* = 0.006; OR: 0.06; 95% CI: 0.008–0.45). **Conclusions:** The findings of this study concerning ATM expression as a biomarker should be interpreted cautiously due to inherent limitations, including its retrospective design, the semi-quantitative nature of immunohistochemistry for ATM assessment, the small sample size with uneven clinical characteristics, and the relatively short follow-up period, which collectively impact generalizability. Despite these limitations, lower ATM expression was associated with better prognosis and pembrolizumab treatment response, suggesting that it may be a valuable biomarker for predicting these factors.

## 1. Introduction

NSCLC is a substantial global health challenge due to its high occurrence and fatality rates [1]. While smoking remains the leading cause, a growing number of cases are being linked to genetic mutations and environmental factors in individuals who have never smoked [2]. NSCLC, the most common type of lung cancer, is the primary cause of cancer-related deaths worldwide, with a disproportionately high incidence in older populations and across various demographic groups [3]. Patient survival is strongly linked to the stage at diagnosis, with early-stage disease having a much better prognosis than advanced cases [4]. Despite therapeutic advancements, the overall five-year survival rate remains unsatisfactory [5]. Immunotherapy, particularly checkpoint inhibitors targeting programmed death 1(PD-1)/programmed death-ligand 1 (PD-L1) and cytotoxic T-lymphocyte antigen 4 (CTLA-4), has significantly altered NSCLC treatment, demonstrating long-lasting responses in some patients and improving survival, especially in advanced stages [6,7].

ATM, encoded by the ATM gene, is a protein kinase involved in DNA damage repair and plays a crucial role in the cellular response to double-strand DNA breaks (DSBs). When the MRE11-RAD50-NBS1 (MRN) complex detects a DSB, it activates ATM, which then phosphorylates downstream proteins to trigger DNA repair, cell cycle checkpoints, or apoptosis [8]. The critical role of ATM lies in preserving genomic integrity by preventing DNA mutations that contribute to tumor formation and progression. Between 0.2% and 0.7% of the population carries pathogenic germline ATM gene variants, particularly those that result in a truncated protein, which increases susceptibility to breast, ovarian, and pancreatic cancer [9]. Disruptions to the ATM gene coding sequence, most notably from truncating mutations, result in the synthesis of unstable ATM proteins, which undergo cellular degradation, leading to marked reductions in protein expression [10]. Lung cancer patients exhibit a slightly elevated rate of inherited ATM gene mutations (up to 1.2–1.9%) compared to the general population, with this increase being most noticeable in lung adenocarcinoma (LUAD) [11,12].

In addition to germline variants, ATM is often somatically mutated in lung tumors. The Cancer Genome Atlas (TCGA) cohorts reveal that ATM is the most frequently mutated DNA damage response gene in NSCLC, with mutation rates around 9% in LUAD and 4% in lung squamous cell carcinoma [13,14]. The pattern of ATM mutations differs from that of common oncogenes; instead of being confined to specific areas, they are dispersed across the entire 150-kilobase gene [15]. The high frequency of germline and somatic ATM gene variants in non-small-cell lung cancer suggests their potential as prognostic biomarkers and/or predictors of therapeutic response.

The prognostic significance of ATM mutations in NSCLC was investigated in two correlation analyses. Ricciuti et al. found that ATM mutations alone did not affect survival or immunotherapy response, though combined ATM and TP53 mutations correlated with improved PFS after immunotherapy [16]. In contrast, Vokes et al. demonstrated that functionally significant ATM mutations were associated with better OS, especially in patients receiving chemoimmunotherapy [17]. While these results differ, both studies suggest potential prognostic associations requiring further validation.

In our study, we aimed to investigate the prognostic and predictive significance of ATM expression in metastatic NSCLC patients treated with pembrolizumab alone or in combination with chemotherapy (CT).

## 2. Materials and Methods

This study, conducted at the Istanbul Medipol University between 1 January 2022 and 30 December 2024, included 49 metastatic NSCLC patients who were over 18 years old, had no actionable driver mutations (e.g., EGFR, ALK, ROS1, BRAF, KRAS, MET, RET, ERBB2, and NTRK1/2/3), and received first-line pembrolizumab monotherapy or pembrolizumab with platinum chemotherapy.

Patients who had tumor tissue blocks with over 100 tumor cells were included for ATM and PD-L1 re-evaluation, but those with poor performance status (ECOG PS 3/4) and lost to follow-up were excluded from analysis. The median time between the collection of tested samples and the start of treatment was 1.2 months (range: 0.3–2.2).

Patients’ clinical data were gathered retrospectively from their medical records. Data on baseline characteristics such as age, gender, smoking history, histopathological type, the stage of disease at diagnosis, their history of curative intent therapy, T stage, the presence of liver, brain, and bone metastases, PD-L1 status, first-line treatment, and ATM score were recorded after obtaining written informed consent from patients or their relatives. PD-L1 expression was determined using the PD-L1 immunohistochemistry (IHC) 22C3 pharmDx assay (Agilent, Santa Clara, CA, USA), and the results were categorized based on the tumor proportion score.

Immunohistochemistry targeting ATM was conducted on whole-tissue sections of biopsies containing tumors, including tru-cut biopsies and tumor samples from resection materials. To achieve this, 2 μm-thick sections were prepared from paraffin-embedded blocks. Automated immunohistochemistry for ATM (using a rabbit polyclonal antibody at a 1:250 dilution, SANTA CRUZ, G12, Santa Cruz, CA, USA) was performed on a BenchMark ULTRA staining instrument (Ventana Medical Systems, Tucson, AZ, USA) for all cases. Antibody clones were detected with the UltraView DAB IHC Detection Kit, following the manufacturer’s instructions. Positive external controls, such as normal large bowel mucosa, were included on each slide, and nuclear staining was considered positive. A pathologist scored the ATM staining manually.

**Figure 1:**
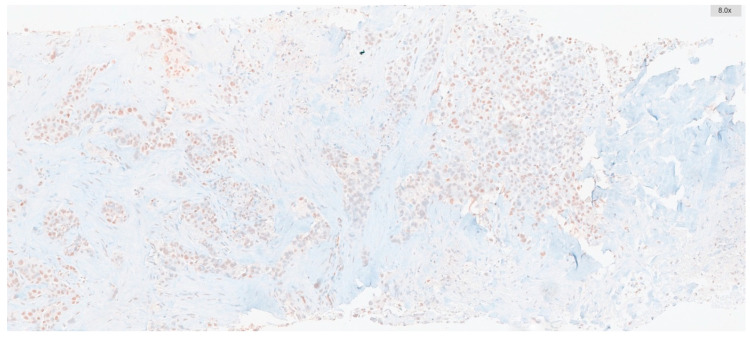
ATM positivity with low expression (1+ nuclear staining is present in 20% of tumor cells).

**Figure 2:**
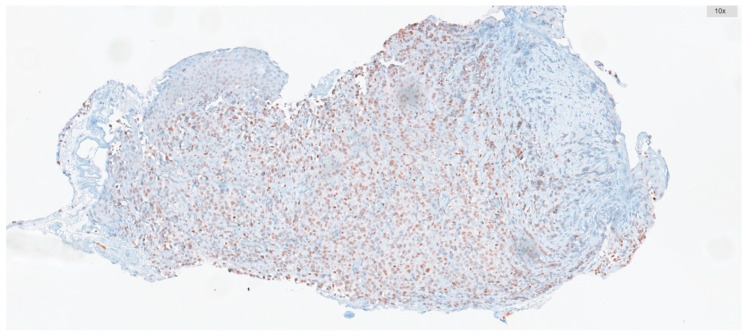
ATM positivity with high expression (2+ nuclear staining is present in 90% of tumor cells).

The percentage of ATM-positive cells and their staining intensities were scored. Intensity was graded as follows: negative, 0; weak, 1; moderate, 2; and strong, 3. The percentage of positive cells was graded as follows: 0, <5%; 1, 5–25%; 2, 26–50%; 3, 51–75%; and 4, >75%. These two measurements were multiplied to obtain weighted scores ranging from 0 to 12, which were used to categorize all cases into low-expression groups (score range: 0–5) and high-expression groups (score range: 6–12) (Figure 1 and Figure 2).

All statistical analyses were conducted using IBM SPSS Statistics 22.0. Descriptive summary statistics for continuous variables are presented as means and standard deviations for normally distributed variables and medians for non-normally distributed variables; frequencies and percentages are given for categorical variables. Chi-squared tests and Fisher’s exact tests were used to compare the relationship between clinicopathological factors and ATM expression. Survival analysis statistics were determined using Kaplan–Meier curves, and the log-rank test was used for comparisons. The prognostic impact of clinicopathological features was evaluated through univariate analysis. PFS was defined as the period from the initiation of therapy to the date of the first documented objective progression of disease, based on Response Evaluation Criteria in Solid Tumors (RECIST 1.1), or death, whichever occurs first, and the time until relapse was defined as the time since diagnosis to the first evidence of relapse. In addition, OS was described as the time from diagnosis to the date of the patient’s death or last known contact. Multivariate analysis was used to assess the significance of ATM expression, and the other clinicopathological prognostic factors were assessed using Cox regression analysis after univariate analysis. The number of events for recurrence and death was 22 and 26, respectively. Hazard ratios (HRs) with 95% confidence intervals (CIs) were calculated and reported. Additionally, multivariate logistic regression analysis was used to identify independent predictors of treatment response. The results are presented as odds ratios (ORs) with 95% CIs. Data are shown as means (SDs), medians (ranges), 95% CIs, or percentages, as applicable. A two-sided *p*-value of <0.05 was considered statistically significant. According to the one-tailed independent samples *t*-test analysis with 95% confidence (1-α), 80% test power (1-β), with a %5% type 1 error level, the number of samples needed for the prediction of ORR was determined to be at least 28 with the G-power program.

Treatment response was assessed using the RECIST (version 1.1) [18]. A complete response (CR) is defined as the disappearance of all target lesions. Any pathological lymph nodes (whether targets or non-targets) must have their short-axis values reduced to <10 mm to be non-pathological. A partial response (PR) is defined as at least a 30% decrease in the sum of diameters of the target lesions, taking the baseline sum diameters as reference values. The ORR was calculated as CR plus PR.

## 3. Results

The mean age of patients was 62.7 ± 9.5, with a majority (63.3%) being over 60 years old. Forty-five patients (91.8%) were male, while four (8.2%) were female. Of these patients, 57.1% (*n* = 28) were current smokers, 32.7% (*n* = 16) were former smokers, and 10.2% (*n* = 5) had never smoked. In terms of histology, adenocarcinoma was found in 75.5% (*n* = 37) of cases, and squamous cell carcinoma was found in 22.4% (*n* = 11). Thirty-nine patients (79.6%) presented with metastatic disease at diagnosis, while ten patients (20.4%) developed metastases post-diagnosis. Only six patients (12.2%) underwent curative surgery initially, as the tumors in 43 (87.8%) patients were initially inoperable. The majority of patients (91.8%) received curative intent chemoradiotherapy. Fifteen patients (30.6%) were staged as T1, 14 (28.6%) as T2, 8 (16.3%) as T3, and 12 (24.5%) as T4. Nine patients (18.4%) had liver metastases, fifteen (30.6%) had brain metastases, and twenty-six (53.1%) had bone metastases. PD-L1 expression was >50% in 26 patients (53.1%), 1–50% in 19 patients (38.8%), and ≤1% in four patients (8.2%). The median number of pembrolizumab cycles was 9 (range: 3–35). Nine patients (18.4%) received pembrolizumab monotherapy, while forty (81.6%) received it in combination with chemotherapy.

Our analysis revealed a significant difference between ATM expression and gender, where women had higher levels of ATM expression compared to men (*p* = 0.04) (Table 1). There were no significant differences between other clinicopathological features and ATM expression. Table 1 summarizes the relationship between patient characteristics and ATM expression.

**Figure 3:**
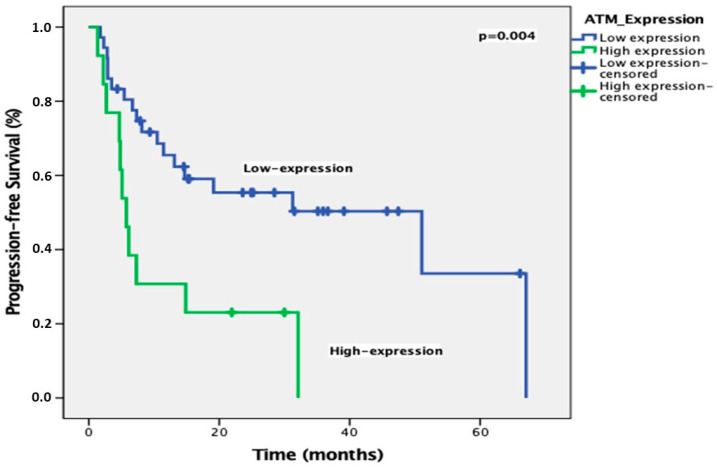
Progression-free survival curves according to the ATM expression levels.

**Figure 4:**
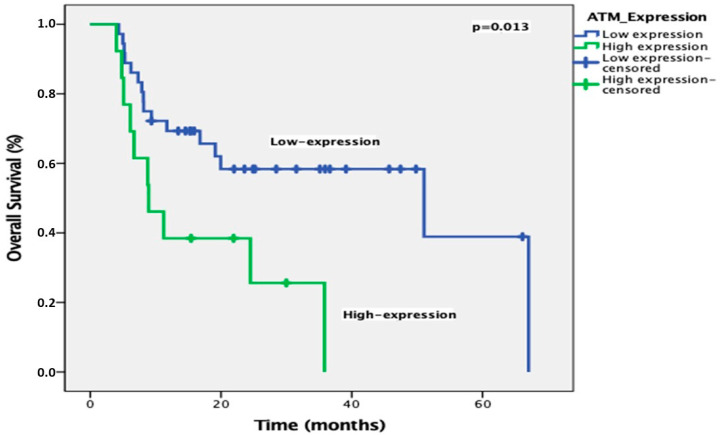
OS curves according to ATM expression levels.

The ORR was 69.4% (CR: 16.3%; PR: 53.1%). ATM expression was high in 37 patients (75.5%) and low in 12 patients (24.5%). Univariate analysis showed that gender and ATM expression were prognostic factors for PFS, while ATM expression was the only prognostic factor for OS (Table 2 and Table 3). With a median follow-up of 25.5 months, the median PFS was significantly longer in patients with low ATM expression compared to those with high expression (51 months vs. 5.7 months, *p* = 0.004) (Figure 3). In multivariate analysis, only ATM expression was an independent prognostic factor for PFS (Table 2). In addition, the median OS was significantly shorter in patients with high ATM expression compared to those with low expression (51 months vs. 8.9 months, *p* = 0.013) (Figure 4). After the multivariate analysis for OS, ATM expression was identified as the only independent prognostic factor (HR 2.41; *p* = 0.034) (Table 3).

Logistic regression analysis revealed that ATM expression is a significant predictor of treatment response in patients with metastatic NSCLC receiving pembrolizumab-based therapy, i.e., patients with high ATM expression had a significantly lower likelihood of responding to treatment (OR: 0.06; *p* = 0.006; 95% CI: 0.008–0.45). In contrast, gender, first-line treatment type, and brain metastases were not associated with treatment response. However, liver and bone metastases were significantly linked to a higher likelihood of treatment response (OR: 26.65, *p* = 0.023, 95% CI 1.58–447 and OR: 10.99, *p* = 0.031, 95% CI 1.25–96.7, respectively) (Table 4). These findings highlight the potential importance of ATM expression as a biomarker for predicting treatment outcomes in NSCLC.

## 4. Discussion

Two large-scale next-generation sequencing (NGS) studies investigated ATM mutations in NSCLC, revealing a significant prevalence of damaging ATM mutations ranging from 9.7% to 11.2% [16,17]. Both studies identified shared associations: ATM mutations correlated with female sex, smoking history, non-squamous histology (adenocarcinoma), high tumor mutation burden (TMB), and PD-L1 positivity. Additionally, these mutations were positively linked to KRAS, STK11, KMT2D, and KEAP1 mutations, while showing inverse correlations with TP53 and EGFR mutations [16,17]. Furthermore, through immunohistochemistry and reverse-phase protein arrays, both research groups confirmed that truncating ATM mutations resulted in reduced ATM protein expression compared to missense mutations [16,17].

Multiple studies have confirmed that NSCLC is often characterized by DNA repair deficiencies caused by ATM mutations [19,20,21]. Two recent investigations found that ATM deficiency in LUAD occurs in 18% to 40% of cases [22,23]. In our study, 75.5% of patients exhibited high ATM expression, suggesting that alterations in ATM are common in NSCLC. Petersen and colleagues identified the ATM expression index (ATM-EI) as a significant prognostic factor for both DFS and OS in stage II/III NSCLC [24]. The authors of the Ricciuti et al. study found a correlation between the complete loss of ATM expression in tumors and a higher likelihood of smoking history [16]. However, our study did not discover a similar association. Patients with low ATM expression exhibited worse survival compared to those with high ATM expression. The effect of ATM expression on survival was more evident in advanced-stage (II/III) NSCLC patients [24]. In our study, patients with low ATM expression had significantly longer PFS compared to those with high expression. In Vokes et al.’s study, ATM mutations were associated with improved survival in patients treated with chemoimmunotherapy [17]. In a primarily first-line setting, Ricciuti et al.’s study did not find a statistically significant difference in treatment outcomes (e.g., ORR, PFS, and OS) based on ATM mutation status in patients receiving PD-(L)1 immune checkpoint blockade therapy combined with platinum doublet chemotherapy [16]. However, there was a numerical trend towards a higher ORR in the ATM mutant group compared to the ATM wild-type group [16]. Our study, using logistic regression analysis, found that ATM expression is a significant predictor of treatment response in patients with metastatic NSCLC receiving pembrolizumab-based therapy. Patients with high ATM expression had a significantly lower likelihood of responding to treatment (OR: 0.06; *p* = 0.006; 95% CI: 0.008–0.45). Our findings are thus compatible with those in the literature [16].

Our findings suggest that ATM expression is a clinically relevant biomarker in NSCLC. Tumors that have ATM mutations or reduced ATM protein levels may exhibit an altered DNA damage response, potentially leading to increased genomic instability and a higher tumor mutation burden (TMB) [8].

This higher TMB can result in the increased production of neoantigens (novel proteins arising from mutations), which may enhance the tumor’s visibility to the immune system and increase its responsiveness to immunotherapies like pembrolizumab.

A loss of ATM function can also affect the expression of molecules on tumor cells that are involved in interactions with immune cells. Because these interactions are crucial for an effective anti-tumor immune response, ATM deficiency may modify them [25].

Furthermore, ATM deficiency might influence the tumor microenvironment, impacting the movement and activity of immune cells, possibly by changing the production rate of cytokines or chemokines that attract or suppress immune cells [26].

Since ATM plays a role in initiating apoptosis (programmed cell death) in response to DNA damage, tumors with ATM alterations may have altered apoptotic pathways [8]. If tumor cells become more resistant to cell death, pembrolizumab’s effectiveness may be reduced.

Assessing ATM expression could aid in identifying patients who may benefit from alternative treatment approaches or targeted therapies. Additional studies are required to validate these results and explore the underlying biological mechanisms linking ATM expression to patient outcomes. Given the limitations of current biomarkers like PD-L1 and TMB, as well as the increasing number of treatment options, additional biomarkers are necessary to effectively guide clinical decisions. These biomarkers may also be important in early-stage treatments like neoadjuvant and adjuvant therapies [27,28,29].

This study has several limitations that should be considered when interpreting the results. First, the retrospective nature of the study introduces potential biases related to patient selection and data collection. Second, ATM expression was assessed using IHC, a semi-quantitative method that can be subject to variability in staining and interpretation. While we took steps to standardize the IHC and scoring procedures, the results may not be as precise as those obtained with more quantitative techniques.

Third, the sample size, particularly the number of patients with low ATM expression (*n* = 12), was relatively small. This limited the statistical power of some of our analyses and may have affected our ability to detect significant differences in certain subgroups. Additionally, the study population had an uneven distribution of some clinical characteristics, such as gender, which further limits the generalizability of our findings.

Our study did not include an analysis of other potentially relevant biomarkers, such as tumor mutational burden (TMB) and lymphocyte densities. These factors are known to influence responses to PD-L1 blockades, and their omission may limit the comprehensiveness of our analysis. Unfortunately, due to financial constraints, we were unable to perform these additional tests, which represents a significant limitation. Finally, the follow-up time in our study, with a median of 25.5 months, may be relatively short for fully evaluating long-term outcomes. Despite this limitation, we believe that our study provides important findings into the potential role of ATM expression as a biomarker in this context and contributes important findings to the literature.

## 5. Conclusions

In conclusion, our study suggests that ATM expression is a valuable biomarker for predicting patient outcomes in metastatic NSCLC treated with pembrolizumab monotherapy in a first-line setting. Further research is necessary to confirm these findings and explore the potential clinical applications of targeting ATM in this patient population. Future studies should aim to validate our findings in larger, prospective cohorts and investigate the underlying mechanisms through which ATM expression affects patient outcomes. Additionally, studies exploring the potential therapeutic benefits of targeting ATM in NSCLC patients with high ATM expression are warranted.

## Figures and Tables

**Table 1:** Tumor and patient characteristics according to ATM expression levels.

Clinicopathological Features	Low ATM Expression *n* (%)	High ATM Expression *n* (%)	*p*-Value
Age, years			0.49
≤60	15 (40.5)	3 (25.0)	
>60	22 (59.5)	9 (75.0)	
Gender			0.04
Female	1 (2.7)	3 (25.0)	
Male	36 (97.3)	9 (75.0)	
Smoking history			0.34
Never	3 (8.1)	2 (16.7)	
Current	20 (54.1)	8 (66.7)	
Former	14 (37.8)	2 (16.7)	
Histopathological type			0.16
Adenocarcinoma	30 (81.1)	7 (58.3)	
Squamous cell carcinoma	6 (16.2)	5 (41.7)	
NOS	1 (2.7)	0 (0.0)	
Initially metastatic			0.23
Yes	31 (83.8)	8 (66.7)	
No	6 (16.2)	4 (33.3)	
Curative surgery			0.62
Yes	4 (10.8)	2 (16.7)	
No	33 (89.2)	10 (83.3)	
Curative CRT			0.25
Yes	2 (5.6)	2 (16.7)	
No	34 (94.4)	10 (83.3)	
T stage			0.39
T1	13 (35.1)	2 (16.7)	
T2	11 (29.7)	3 (25.0)	
T3	6 (16.2)	2 (16.7)	
T4	7 (18.9)	5 (41.7)	
Liver metastases			0.66
Present	6 (16.2)	3 (25.0)	
Absent	31 (83.8)	9 (75.0)	
Brain metastases			0.73
Present	12 (32.4)	3 (25.0)	
Absent	25 (67.6)	9 (75.0)	
Bone metastases			1.00
Present	20 (54.1)	6 (50.0)	
Absent	17 (45.9)	6 (50.0)	
PD-L1 status (TPS)			0.90
<1%	3 (8.1)	1 (8.3)	
1–50%	15 (40.5)	4 (33.3)	
>50%	19 (51.4)	7 (58.3)	
First-line treatment			1.00
Pembrolizumab monotherapy	7 (18.9)	2 (16.7)	
Pembrolizumab plus platinum doublets	30 (81.1)	10 (83.3)	

PD-L1: programmed death-ligand 1; TPS: tumor proportion score; ATM; ataxia–telangiectasia mutated; CRT: chemoradiotherapy.

**Table 2:** Univariate and multivariate analysis results for PFS.

Variable	Median PFS (Months)	Univariate *p*-Value	HR (95% CI)	Multivariate *p*-Value
Age, years		0.23	2.08 (0.75–4.48)	0.13
≤60	31.2			
>60	10.5			
Gender		0.03	0.33 (0.06–1.68)	0.18
Female	6.1			
Male	31.2			
Initially metastatic		0.44	0.75 (0.16–3.34)	0.70
Yes	13.1			
No	19.3			
Curative surgery		0.19	0.59 (0.10–2.77)	0.62
Yes	51.0			
No	11.4			
T stage		0.44	0.54 (0.12–2.26)	0.39
T1	51.0			
T2	14.8			
T3	8.1			
T4	5.4			
Liver metastases		0.26	0.54 (0.12–2.45)	0.43
Present	10.5			
Absent	19.1			
Bone metastases		0.11	2.95 (1.14–4.64)	0.02
Present	11.4			
Absent	51.0			
PD-L1 status (TPS)		0.66	0.87 (0.38–1.99)	0.74
<1%	8.13			
1–50%	13.1			
>50%	19.1			
First-line treatment		0.15	2.76 (0.67–6.42)	0.15
Pembrolizumab monotherapy	51.0			
Pembrolizumab plus platinum doublets	13.1			
ATM score		0.004	1.98 (1.12–4.56)	0.039
Low expression	51.0			
High expression	5.7			
Site of metastasis		0.38	0.84 (0.45–1.55)	0.58
Liver	7.3			
Brain	32.1			
Bone	11.4			

PD-L1: programmed death-ligand 1; TPS: tumor proportion score; HR: hazard ratio; PFS: progression-free survival; ATM: ataxia–telangiectasia mutated.

**Table 3:** Univariate and multivariate analysis results for OS.

Variable	Median OS (months)	Univariate *p*-Value	HR (95% CI)	Multivariate *p*-Value
Age, years		0.29	1.85 (0.62–5.48)	0.26
≤60	35.8			
>60	19.1			
Gender		0.16	0.42 (0.08–2.24)	0.31
Female	8.1			
Male	35.8			
Initially metastatic		0.42	1.94 (0.29–3.42)	0.49
Yes	19.9			
No	51.0			
Curative surgery		0.30	1.82 (0.14–4.23)	0.64
Yes	51.0			
No	19.9			
T stage		0.13	0.42 (0.11–1.57)	0.20
T1	51.0			
T2	67.0			
T3	19.1			
T4	7.9			
Liver metastases		0.81	0.45 (0.09–2.31)	0.34
Present	35.8			
Absent	24.5			
Bone metastases		0.55	1.79 (0.67–4.77)	0.24
Present	19.9			
Absent	51.0			
PD-L1 status (TPS)		0.59	0.92 (0.42–2.04)	0.84
<1%	9.36			
1–50%	35.8			
>50%	19.1			
First-line treatment		0.30	3.96 (0.89–6.16)	0.07
Pembrolizumab monotherapy	51.0			
Pembrolizumab plus platinum doublets	24.5			
ATM score		0.013	2.41 (0.86–5.37)	0.034
Low expression	51.0			
High expression	8.9			
Site of metastasis		0.90	1.06 (0.55–2.04)	0.86
Liver	NR			
Brain	35.83			
Bone	19.96			

PD-L1: programmed death-ligand 1; TPS: tumor proportion score; HR: hazard ratio; PFS: progression-free survival; ATM: ataxia–telangiectasia mutated; NR: not reached.

**Table 4:** Predictors of first-line pembrolizumab response in metastatic NSCLC.

Factors	Coefficient ß	Wald X^2^	*p*	OR	95% CI
ATM score (low vs. high)	−2.80	7.42	0.006	0.06	0.008–0.45
Gender	−0.14	0.003	0.95	0.86	0.007–109
First-line treatment	−1.74	2.12	0.14	0.17	0.017–1.81
Liver metastasis	3.28	5.20	0.023	26.65	1.58–447
Brain metastasis	1.47	1.97	0.16	4.38	0.55–34.5
Bone metastasis	2.39	4.67	0.031	10.99	1.25–96.7

## Data Availability

The data supporting this study’s findings are not openly available. Further enquiries can be directed to the corresponding author.
